# Long-Term Analysis of Internal Exposure Dose-Reduction Effects by Food Regulation and Food Item Contribution to Dose after the Fukushima Daiichi Nuclear Power Plant Accident

**DOI:** 10.3390/foods12061305

**Published:** 2023-03-18

**Authors:** Minoru Osanai, Mayu Miura, Chihiro Tanaka, Kohsei Kudo, Shota Hosokawa, Megumi Tsushima, Tomuhiro Noro, Kazuki Iwaoka, Masahiro Hosoda, Ichiro Yamaguchi, Yoko Saito

**Affiliations:** 1 Department of Radiation Science, Hirosaki University Graduate School of Health Sciences, Hirosaki 036-8564, Japan; 2 Department of Radiological Technology, Hirosaki University School of Health Sciences, Hirosaki 036-8564, Japan; 3 Department of Radiation Regulatory Science Research, National Institute of Radiological Sciences, National Institutes for Quantum Science and Technology, Inage 263-8555, Japan; 4 Department of Environmental Health, National Institute of Public Health, Wako 351-0197, Japan

**Keywords:** food safety, food regulation, risk assessment, internal exposure dose, radioactive materials, standard limits, food monitoring test, Fukushima Daiichi Nuclear Power Plant accident, dietary intake, food consumption

## Abstract

Over 10 years have passed since the Fukushima Daiichi Nuclear Power Plant accident. This study verifies the efficacy of longitudinal regulation on internal exposure doses and analyzes food group contributions to radiation doses using accumulated monitoring test results. The committed effective doses in 10,000 virtual persons from fiscal year (FY) 2012 to 2021, with and without regulation, were estimated as products of radioactivity concentrations randomly sampled from the test results, food intake, and dose coefficient. The distributed values of food intake rather than a mean value in dose estimation were assumed to reflect food intake variations and avoid underestimation of internal exposure doses for high-intake consumers. Furthermore, the ingestion of radioactive cesium from the calculation was analyzed per food group. The 95th percentile of the internal exposure dose (the dose of a “representative person”) was less than 1 mSv/year in both FYs. The regulation effect was substantial in FY 2012, and no noticeable difference in radiation doses was found between the regulation and no regulation conditions after FY 2016. Internal exposure doses decreased until approximately FY 2016 and then remained constant. It was also shown that not only radioactivity concentration but also food intake is a major factor affecting cesium intake. In summary, it was confirmed that Japan had ensured food safety regarding radioactive materials.

## 1. Introduction

More than 10 years have passed since the Fukushima Daiichi Nuclear Power Plant (FDNPP) accident in 2011. The provisional regulation value for radioactive cesium based on 5 mSv/y as an effective dose was established after the accident as a food safety measure [1,2,3,4,5]. Following the provisional regulation value, new criteria for radioactive cesium (sum of ^134^Cs and ^137^Cs) based on 1 mSv/y as the effective dose were established in fiscal year (FY) 2012 [3,4,5,6,7,8,9] and are still being applied. Table 1 shows the values of the current criteria. As a side note, the provisional regulation values and current criteria were established for dominant radionuclides (i.e., radioactive cesium) considering the radiation dose contributions from radionuclides other than radioactive cesium, such as strontium-90 [3,4,5,6,8,10,11].

Monitoring tests are conducted throughout Japan according to the current criteria, primarily in 17 eastern prefectures, including Fukushima Prefecture [8,12]. Foodstuffs that exceed the current criteria are collected and discarded. If food items that exceed standard values are spread regionally, their distribution is restricted in the affected regions [8,12,13]. Thus, Japan has taken rigorous measures to prevent the distribution of foods that exceed the standard limits.

Numerous monitoring tests have been conducted based on the provisional regulation values or current criteria, and the Ministry of Health, Labour, and Welfare (MHLW) and other related organizations [8,14,15] have released the results. More than 2,700,000 samples from the postaccident period to FY2021 were included in the monitoring tests. We have been effectively using these accumulated monitoring test results and verifying the effectiveness of the food regulations after the FDNPP accident in Japan [16,17]. However, the types of foods that contribute to radiation exposure dose have not been specified. In order to ensure the radiation safety of the population, it is helpful to identify the contribution of individual food groups to the radiation dose. In addition, as in the case of the Chernobyl nuclear power plant accident, the effects on food may persist over the long term, so it is essential to verify food safety continuously. In this study, the period targeted for verification was significantly extended, and the dose contribution ratio of each food category was analyzed. Furthermore, although previous studies have used food intake as an average (i.e., fixed) value to estimate internal doses, this study attempted a method considering food intake distribution that would not underestimate the exposure doses of high-intake individuals. This study aimed to verify the efficacy of longitudinal regulation on internal exposure doses after the FDNPP accident and analyze food group contributions to radiation doses, taking care not to underestimate the radiation dose. If food safety with respect to radioactive materials is objectively demonstrated through this study, it would reassure anxious local residents and people all over the world.

## 2. Materials and Methods

The basic methodology is in accordance with our previous studies [16,17]. On this basis, the target year was expanded, the radiation dose estimation method was further refined, and the food categories contributing to the radiation dose were analyzed.

### 2.1. Subject for Estimation

The radiation exposure dose caused by food ingestion was evaluated for 10 years (FY2012–FY2021) after the current criteria were applied, using monitoring test results from across Japan.

### 2.2. Data Preparation

#### 2.2.1. Results of Monitoring Inspection

The monitoring test results in the report on the MHLW homepage [14] were obtained. The activity concentration of each sample (^134^Cs, ^137^Cs, and the sum of ^134^Cs and ^137^Cs) (Bq/kg) is shown with the purchase or sampling day, the rough food categories, the food item names (6670 items for 10 years), and the production area. In this study, the monitoring test results for each FY were classified by purchase or sampling day, and the total radioactivity was used to estimate radiation dose. Results for which the total activity concentration, purchase day, or sampling day were not indicated were excluded. The database categorized by FY was structured with cleaned monitoring test results by Microsoft ACCESS 2019 (Microsoft Japan Co., Ltd., Tokyo, Japan). Furthermore, the radioactivity concentrations of the monitoring test results by food category (100 classifications described in the following section) were analyzed.

#### 2.2.2. Food Intake

The food consumption weight for adults (men and women older than 20 years) reported in the 2012 National Health and Nutrition Survey (NHNS), Japan, was used [18]. This survey shows the average food intake (g/day) classified into 98 small categories of foodstuffs (Table 2) [19]. The average food intake value is typically used in radiation dose estimation studies of internal exposure due to food ingestion [20], including in our previous reports [16,17]. However, radiation dose estimation based on average food intake might underestimate internal doses for people with high intake. Therefore, this study proposes a method that considers food intake distribution to reflect the variation in food intake among individuals. Because the NHNS, Japan, reports the mean, standard deviation (SD), and median of food intake, this study assumed a normal distribution for food intake as a simple model using the mean value and SD. The food intake dataset for each classification was obtained using the Monte Carlo method. The random module is a built-in module of Python version 3.11 (Python Software Foundation, Dover, DE, USA) that is used to generate pseudo-random numbers. The intake for each food classification and each virtual person was obtained by passing the mean and SD values to the argument of the Gauss method in the random module. If a negative value was produced, retries were performed until a positive value was obtained. A food intake dataset for 100,000 virtual persons was obtained for each food category. The number of virtual persons was determined to reduce variation in the process of generating food intakes in the random module.

Because drinking water intake is excluded in the NHNS, Japan, it was set as 2 L/day based on the same assumptions as when the current standard limits were derived [3,21]. Therefore, because an intake distribution for drinking water could not be assumed, it was fixed.

Furthermore, wild vegetables have diverse values for residents, for example, picking, consuming, selling, sharing, and preserving [22]. However, wild vegetables are known as foodstuffs that can contain moderately high levels of radioactive cesium [8,23,24,25,26]. Similar to drinking water, the intake of wild vegetables is unspecified in the NHNS, Japan. Therefore, we selected foods that would be classified as wild vegetables from the results of a detailed food intake survey irregularly conducted in 2010 [27] and totaled their intakes. As in our previous study [17], the summed value of 7.67 g/day was used as the wild vegetable intake. Because the value is the sum of the average individual wild vegetable intake, no SD (i.e., variation in intake among individuals) information is available. However, because wild vegetable consumption is likely to vary among individuals, it is essential to consider the intake volume distribution to avoid underestimating the internal exposure dose in high intake. Hence, in this study, the SD for wild vegetable intake was determined based on the ratios of the SD to the mean vegetable intake (small classification: 25 to 38) in the NHNS, Japan. Specifically, the maximum value of the SD to the mean of vegetable intake ratios was 4.3 (range: 1.1–4.3). Therefore, the SD of 38 g/day was determined for wild vegetables by multiplying the wild vegetable intake (7.67 g/day) by 5.0 (the maximum value of 4.3 was rounded up to 5.0).

**Table 2:** The mean value and standard deviation (SD) of food intake in each food category. Numbers 1–98 indicate the number of small classifications in the National Health and Nutrition Survey (NHNS) [18,19].

No.	Small Classification	Intake (g/Day)		No.	Small Classification	Intake (g/Day)		No.	Small Classification	Intake (g/Day)	
		Mean	SD			Mean	SD			Mean	SD
1	Rice	328	186	35	Other vegetables	48.3	55	69	Other meats and Processed products	0.00898	0.46
2	Rice products	4.18	25	36	Vegetable juices	12.7	54	70	Eggs	34.1	34
3	Wheat flour	3.56	12	37	Leaf pickles	3.82	16	71	Milk	60.6	103
4	Breads (except Japanese buns)	33.1	44	38	Other pickles	8.44	19	72	Cheeses	2.48	7.9
5	Japanese buns	4.39	22	39	Strawberries	0.0889	2.9	73	Fermented milk and Lactic acid bacteria beverages	30.6	59
6	Japanese noodles and Chinese noodles	43.0	88	40	Citrus fruits	22.7	55	74	Other dairy products	6.18	37
7	Precooked noodles	5.13	22	41	Bananas	15.9	39	75	Others (in Milks)	0	0
8	Macaroni and Spaghetti	10.7	46	42	Apples	21.9	52	76	Butters	0.999	3.0
9	Other wheat products	5.38	20	43	Other fruits	39.9	82	77	Margarines	1.25	3.4
10	Buckwheat and Buckwheat products	6.52	39	44	Jams	1.29	5.0	78	Vegetable fats and oils	8.05	8.3
11	Corn and Corn products	0.388	5.3	45	Fruit juices and Fruit juice beverages	8.03	48	79	Animal fats	0.108	0.86
12	Other cereals	2.04	20	46	Mushrooms	17.2	29	80	Others (in Fats and Oils)	0.00779	0.25
13	Sweet potatoes and Sweet potato products	6.97	27	47	Algae	10.5	21	81	Traditional confectioneries	11.7	29
14	Potatoes and Potato products	25.7	47	48	Horse mackerels and Sardines	9.14	28	82	Cakes, Buns, and Pastries	6.71	24
15	Other potatoes and Potato products	19.8	41	49	Salmons and Trout	5.54	21	83	Biscuits	1.62	8.5
16	Starches and Starch products	1.98	8.5	50	Sea breams and Righteye flounders	5.69	23	84	Candies	0.165	2.1
17	Sugars and Sweeteners	6.74	8.5	51	Tunas, Marlins, and Swordfishes	4.77	21	85	Others (in Confectioneries)	4.67	20
18	Soybean (whole beans) and its products	1.28	8.1	52	Other fishes	9.02	29	86	Sake	11.3	54
19	*Tofu* (Bean curd)	35.5	57	53	Shellfishes	3.16	15	87	Beer	76.7	225
20	*Abura-age*	8.18	22	54	Cephalopods	4.21	17	88	Wines, Spirits, and Others	36.1	138
21	*Natto* (Fermented soybeans)	8.04	17	55	Prawns, Shrimps, and Crabs	4.73	18	89	Teas	296	358
22	Other soybean products	7.20	39	56	Seafood (salted, semi-dried, and dried)	15.8	32	90	Coffees and Cocoas	151	198
23	Other pulses and Pulse products	1.40	9.0	57	Seafood (canned)	2.31	11	91	Others (in Other beverages of Beverages)	102	228
24	Nuts and Seeds	2.24	8.5	58	Seafood (*Tsukudani*)	0.294	3.2	92	Sauces	1.88	5.6
25	Tomatoes	15.2	37	59	Seafood (Fish paste products)	10.2	26	93	*Shoyu*: soy sauces	14.2	14
26	Carrots	20.3	27	60	Fish hams and Sausages	0.729	6.5	94	Edible salts	1.35	1.5
27	Spinach	14.7	35	61	Beefs	14.5	35	95	Mayonnaise	2.89	6.1
28	Sweet peppers	4.86	14	62	Pork	33.7	46	96	Miso	11.6	12
29	Other green and yellow vegetables	35.9	52	63	Hams and Sausages	12.6	22	97	Other seasonings	63.4	88
30	Cabbages	28.6	49	64	Other animal meats	0.361	8.3	98	Spices and Others	0.333	1.1
31	Cucumber	9.68	21	65	Chickens	23.9	46		Drinking water ^1^	2000	-
32	*Daikon* (Japanese radishes)	32.4	56	66	Others (in Poultries of Meats)	0.0751	2.8		Wild vegetables ^2^	7.67	38
33	Onions	31.4	42	67	Offal	1.52	13				
34	Chinese cabbage	20.4	50	68	Whale meat	0.0354	1.5				

^1^ The intake volume of drinking water, which is not recorded in the NHNS, Japan, was determined to be a fixed value of 2 L/day. ^2^ The intake weight of each wild vegetable was obtained from a survey [27] other than the NHNS, Japan, and summarized. The SD for wild vegetables (i.e., 38 g/day) was determined based on the mean and SD in the vegetable category.

As described above, a total of 100 food categories, including drinking water and wild vegetables, were considered in this study for radiation dose estimation.

As in our previous study [16,17], the foodstuff item names (6670 items in 10 years) in the monitoring test results were corresponded to the 100 categories in the food intake data. Our previous study described the methodology in detail [16].

#### 2.2.3. Dose Coefficient for Dose Calculation

The dose coefficient (*DC*) (Sv/Bq) based on the International Commission on Radiological Protection (ICRP) publication 72 [28] was used to estimate radiation dose. The ICRP provides *DC*s for the ingestion of each radionuclide. Because the sum radioactivity of ^134^Cs and ^137^Cs was used in this study to calculate radiation exposure dose, the *DC*s for the total activity of ^134^Cs and ^137^Cs were determined. *DC*s of 1.9 × 10^−8^ and 1.3 × 10^−8^ for ^134^Cs and ^137^Cs for an adult, respectively, are provided. The physical half-lives of ^134^Cs and ^137^Cs are 2.06 and 30.2 years, respectively [29]. We calculated the weighted average *DC*s for the total activity of ^134^Cs and ^137^Cs for each FY using a decreasing rate based on the half-lives of ^134^Cs and ^137^Cs using the following formula:

(1) $$Weighted\;average\;DC\;(Sv/Bq)=(1.9\cdot 10^{-8}{\cdot DR}_{134,y}+1.3\cdot 10^{-8}{\cdot DR}_{137,y})/({DR}_{134,y}+{DR}_{137,y})$$

where *DR*_134,y_ and *DR*_137,y_ are the decay rates of ^134^Cs and ^137^Cs in each FY, respectively. The decreasing rate in each FY was calculated using the physical half-life and lapsed time. Table 3 lists the weighted average *DC*s for each FY.

### 2.3. Data Sampling and Radiation Dose Calculation

**Figure 1:**
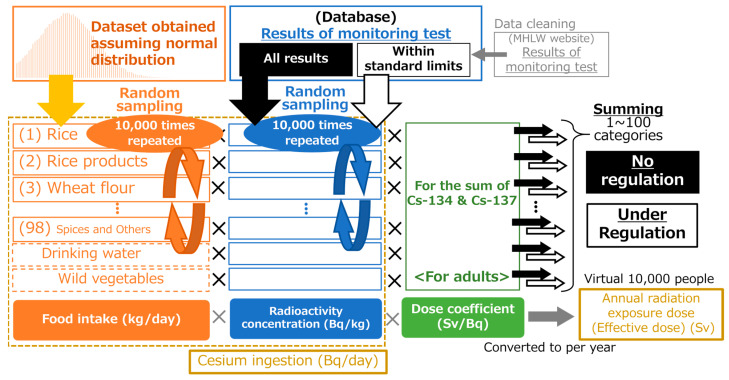
Summary of the dose estimation procedure. First, repeated random sampling of food intake was conducted for every food classification. Next, random sampling for the monitoring inspection results was repeated within limits or for all results. The internal exposure dose of 10,000 persons was calculated as the product of the food intake, the radioactivity concentration of monitoring test results, and the dose coefficient. The number in parentheses in front of the food category name represents a small classification number in the National Health and Nutrition Survey (NHNS), Japan.

Figure 1 summarizes the dose estimation procedure used in this study. The radiation dose for 10,000 virtual persons was estimated under the assumption of with and without food regulation. First, random sampling of food intake was repeated 10,000 times for every 100 food classifications. The same food intake dataset obtained by random sampling was used for dose calculation for each FY. Next, the monitoring test results (i.e., radioactivity concentration in food) were randomly sampled per food classification for each FY. A random sampling of monitoring test results was also repeated 10,000 times for every food classification in the following two cases: (i) the internal exposure dose in the no-regulation condition was assessed using all inspection results, and (ii) the internal exposure dose under the regulation condition was assessed using the monitoring test results within standard limits. This methodology assumed that no food above the standard limits was consumed under the regulation condition. A similar methodology to estimate the internal exposure dose under food regulation was conducted previously using monitoring test results within standard limits when the current criteria for radionuclides in foods were developed by the MHLW [3,30]. In this study, because both the amount of food intake and the radioactivity concentration were obtained by random sampling, this method is generally considered a “probabilistic estimation method” among radiation dose estimation methods [30]. A random sampling system (K2 Computing, Owani, Japan) was programmed with Structured Query Language and Visual Basic for Applications (Microsoft Japan Co., Ltd., Tokyo, Japan) using Microsoft ACCESS 2019 (Microsoft Japan Co., Ltd., Tokyo, Japan) as a platform. The number of random samplings was determined considering the variation due to the use of random numbers based on previous experience (i.e., variation is adequately small at 10,000 repetitions).

The committed effective dose as the internal exposure dose was calculated by multiplying the randomly sampled radioactivity concentration in each FY by the randomly sampled food intake volume:

(2) $$Committed\;effective\;dose\;(mSv/year)=365.24\cdot 10^{3}\cdot DC\sum_{i=1}^{100}I_{i}\cdot C_{i}$$

where *I_i_* denotes the randomly sampled food intake in FY 2012 (kg/day) in each food classification, and *C_i_* denotes the radioactivity concentration of radioactive cesium (sum of ^134^Cs and ^137^Cs) (Bq/kg) in each FY sampled randomly in each food classification. *DC* represents the dose coefficient for the sum of ^134^Cs and ^137^Cs (Sv/Bq) in each FY.

Multiplying the radioactivity concentration and food intake was individually performed for each food category and then summed to obtain the total radiation dose for each virtual person.

When the monitoring test result was below the limit of detection (LOD) (i.e., not detected [ND]), the radioactivity concentration was set based on the ND sample ratio in each food category to obtain the radiation exposure dose. This concept correlates with that reported in previous studies [3,5,16,17]. Specifically, the radioactivity concentration was set as the LOD value if the ND ratio was less than 60% in each food category. When the ND percentage was between 60% and 80%, the value was set to half of the LOD; when the ND percentage was greater than 80%, the value was set to one-quarter of the LOD. Furthermore, the radioactivity concentrations of the monitoring test results were adjusted for some food items to assume the condition of consumption (i.e., ready to eat—a concept expressed by the Codex Alimentarius Commission) [31,32]. Based on previous studies, the activity concentration for brown rice was assumed to be a quarter [5,33]. Additionally, for leaves of plants that will be extracted for drinking (other than green tea leaves), the radioactivity concentration was adjusted to one-fiftieth based on a previous study [34]. If a food classification had no test results, the radioactivity concentration was assumed to be 0 Bq/kg.

The breakdown of cesium ingestion (Bq/day) by food classification, obtained when calculating the internal exposure dose (Sv), was also analyzed.

### 2.4. Data Analysis

The median and 95th percentile for analyzed radioactivity concentration in the monitoring test results, the estimated radiation exposure dose, analyzed cesium ingestion volume, and food intake weight are reported and discussed.

## 3. Results

### 3.1. Breakdown of Monitoring Test Results by Food Classification

**Figure 2:**
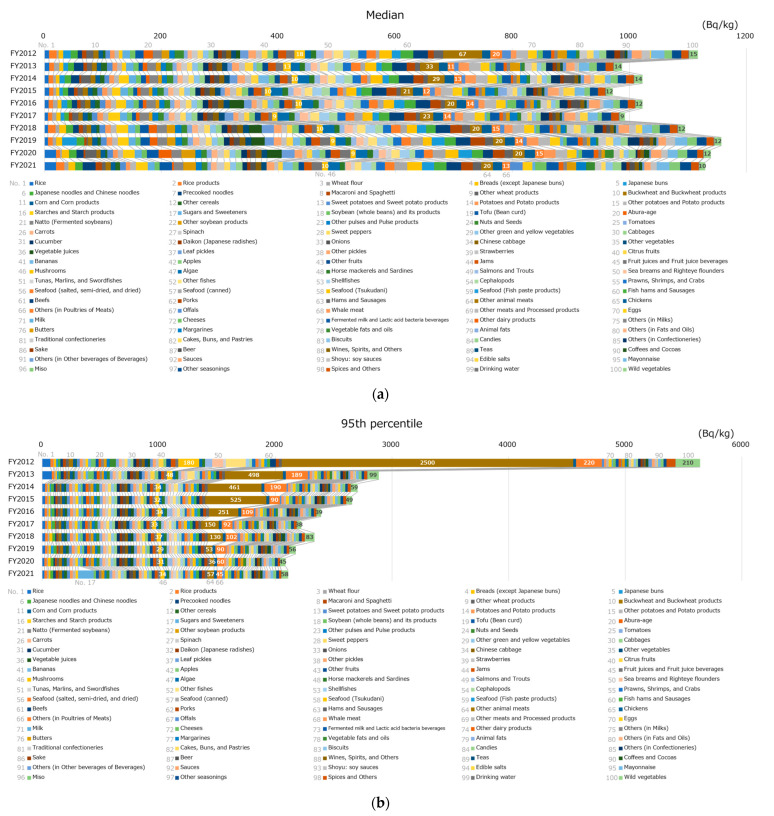
The breakdown of monitoring test results per food classification. The percentile values of radioactivity concentration in each food category were obtained, and the values for all food categories were summarized for each percentile: (**a**) the median and (**b**) the 95th percentile. Results below the limit of detection (LOD) were analyzed as the activity concentration of the LOD. The numbers of the radioactivity of mushrooms, other animal meats, others (in poultries of meats), and wild vegetables are also indicated in the figure as food categories high in radioactivity levels.

Figure 2 shows the breakdown of the radioactivity concentration of the monitoring test results per food classification. Figure 2a,b illustrate the breakdown in the median and the 95th percentile, respectively. There were no significant differences in the radioactivity concentration of each food classification at the median. The value for other animal meats, including wild boar, was slightly higher, especially in FY 2012. However, at the 95th percentile, there were significant differences in the radioactivity concentrations among food classifications. In FY 2012, the largest classification was other animal meats, followed, in order, by others (in poultries of meats), which includes wild birds, wild vegetables, mushrooms, and other fishes. In the following FY, a similar trend was observed for the top four food classifications (i.e., other animal meats had the highest concentration, followed by others [in poultries of meats], wild vegetables, and mushrooms). However, the concentration values decreased significantly compared with those in FY 2012. As an exception, in FY 2021, the radioactivity concentrations in the 95th percentile of sugars and sweeteners were the highest.

### 3.2. Trend of Estimated Radiation Exposure Dose

**Figure 3:**
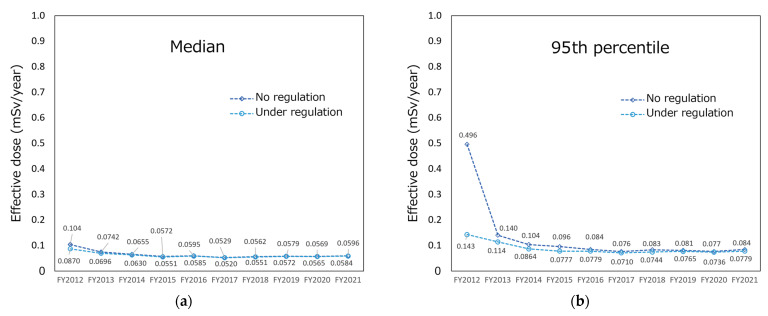
Trend of internal radiation exposure dose in no regulation and under regulation: (**a**) median and (**b**) 95th percentile in the radiation exposure doses of 10,000 virtual persons.

Figure 3 shows the trend of the internal radiation exposure dose of no regulation or under regulation. In addition to the median value in an effective dose of 10,000 virtual persons (Figure 3a), the 95th percentile of a radiation dose (Figure 3b), defined by the ICRP as a radiation dose of a representative person [35], is also shown. In the median and 95th percentile, the effective dose was less than 1 mSv/year in all FYs with no regulation and under regulation. In the median, the radiation dose under regulation was slightly smaller (16.0%) than that of no regulation in FY 2012. Moreover, there were no significant differences (differences were <10%) between no regulation and under regulation in the following years. In the 95th percentile, the effective dose under regulation was more than 10% smaller than that of no regulation from FY 2012 to FY 2015. The difference was significant in FY 2012, as the effective dose under regulation was more than 70% smaller than that of no regulation. After FY 2016, there was no significant difference in the effective dose with no regulation and under regulation—the difference was less than 10%. Overall (no regulation and under regulation, and median and 95th percentile), radiation doses decreased until approximately FY 2016, after which they remained unchanged at low doses.

### 3.3. Breakdown of Cesium Ingestion by Food Classification

**Figure 4:**
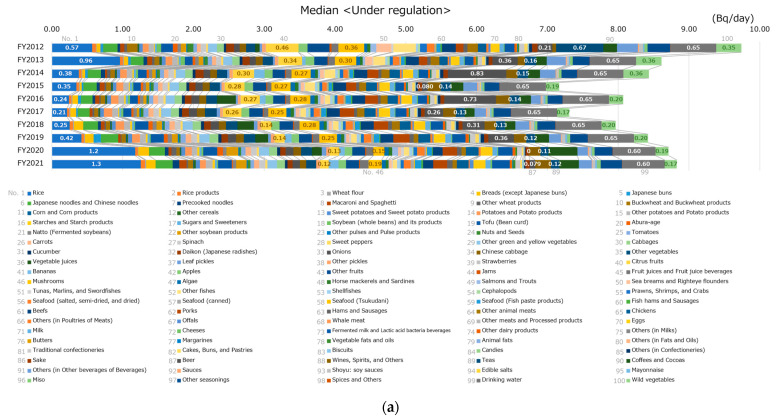

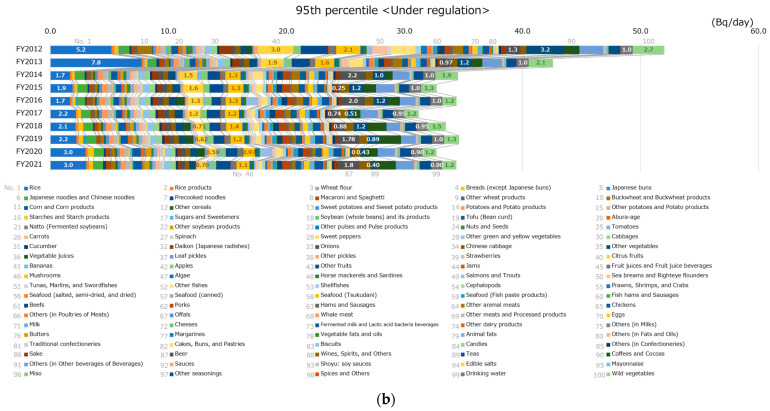
Breakdown of cesium ingestion per food classification: (**a**) median and (**b**) 95th percentile of cesium ingestion by 10,000 virtual persons. The percentile values of cesium ingestion in each food category were obtained, and the values for all food categories were summarized for each percentile. Hence, it does not necessarily mean that the same virtual person ingests them all. For food classifications with moderately high cesium ingestion, the values of cesium ingestion are also indicated in the figure. Specifically, values for rice, citrus fruits, mushrooms, beer, teas, drinking water, and wild vegetables are included.

Figure 4 shows the breakdown of cesium ingestion per day under regulation per food classification. The median and 95th percentile values for cesium ingestion of 10,000 virtual persons are shown in Figure 4a,b, respectively. In the median, there was no category with a notably high intake; however, the drinking water intake was high. In some FYs, cesium ingestion was higher in rice, teas, and beer. Similarly, in the 95th percentile, no category had a notably high cesium intake; however, cesium ingestion in rice was moderately high in some FYs. Cesium ingestion (95th percentile) was the largest in FY 2012 and FY 2013, and it decreased in FY 2014, after which there were no significant differences between the years. Even in FY 2012, the year of maximum cesium ingestion, cesium ingestion per day was less than 100 Bq (the standard limit for general foods is 100 Bq/kg).

### 3.4. Breakdown of Food Intake by Food Classification

**Figure 5:**
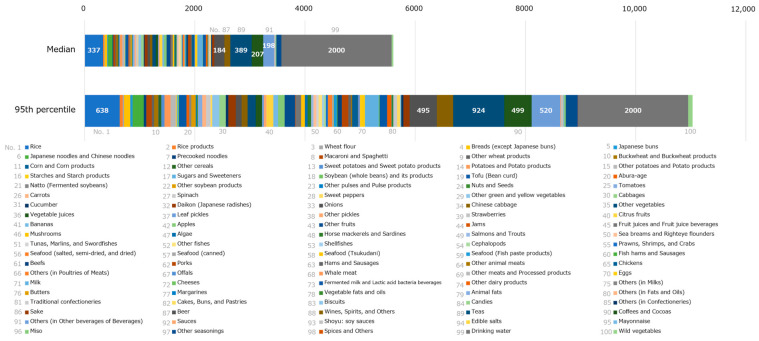
The food intake for each classification was obtained based on the normal distribution assumed with the average value and standard deviation in the 2012 National Health and Nutrition Survey (NHNS), Japan. The food intake percentile values in each food classification were obtained, and the values for all food categories were summarized for each percentile. Hence, this does not necessarily mean that the same person consumes them all in the same proportions. Because the distribution of drinking water intake could not be assumed, it was treated as a fixed value.

Figure 5 shows the food intake volume for each classification. The value was obtained based on a normal distribution assumed with the average and SD. In the median, the food intake was greater for drinking water, teas, and rice, in that order. In the 95th percentile, the food intake was greater for drinking water, teas, rice, others (in other beverages of beverages), coffees and cocoas, and beer, in that order.

## 4. Discussion

General diet studies on radioactive materials do not provide a detailed breakdown of foods that contribute to internal radiation exposure. For instance, in the duplicate diet method, the entire meal is a sample; therefore, the radioactivity concentration of individual foodstuffs cannot be obtained [36]. In a market basket study conducted in Japan, data about the dose contribution of each detailed food category were not provided because the radioactivity concentrations were measured for samples divided into 14 food groups as a rough classification [37,38]. This study focused on the accumulated monitoring test results obtained for individual foodstuffs, analyzed the contribution of each food group to the radiation dose, and verified the dose-reduction effect of food regulations. Furthermore, for food intake, which is necessary for radiation exposure dose calculations, a new method considering the distribution rather than an average value was attempted to avoid underestimating the high-intake internal exposure doses.

**Figure 6:**
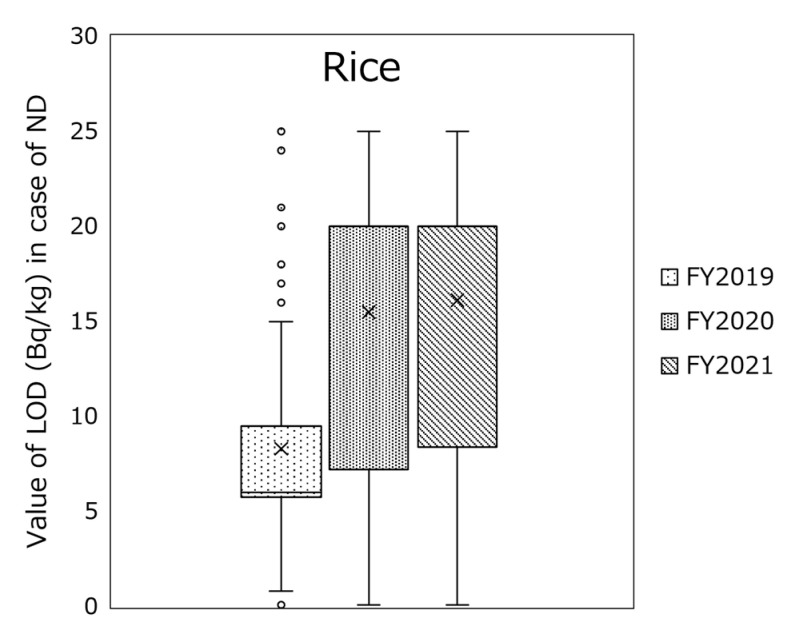
Box plot of the limit of detection (LOD) value in the case of not detected (ND) for rice in fiscal years (FYs) 2019–2021.

The breakdown of radioactivity concentrations of the monitoring test result per food classification shows no significant differences in each food classification at the median (the concentrations of other animal meats in FY 2012 were slightly higher). However, in the 95th percentile, the activity concentrations in other animal meats, others (in poultries of meats), wild vegetables, mushrooms, and other fishes were higher than in other food classifications in FY 2012. It is widely known that radioactivity levels of foodstuffs that cannot be cultivated or farmed under controlled conditions are sometimes high. The overall radioactivity concentrations have decreased considerably. In recent years, differences in food classification have disappeared, and the effect of the FDNPP accident has decreased. However, in FY 2021, the radioactivity concentrations in the 95th percentile of sugars and sweeteners were the highest. They were traced back to a specific food, i.e., honey, with the highest radioactivity level that exceeded the standard limit of 100 Bq/kg, and it was subject to a voluntary recall [39], indicating that safety measures functioned efficiently. A notable finding was that the rice radioactivity concentrations in FYs 2020 and 2021 at the median were higher than those in the past (Figure 2a). We also analyzed this finding and found that the LOD value was a contributing factor. Figure 6 shows LOD values in the case of ND for rice in FYs 2019–2021, indicating that monitoring tests in FY 2019 were conducted with a small LOD (median: 6.0 Bq/kg) and the tests in FYs 2020 and 2021 were conducted with a large LOD (median for both: 20 Bq/kg). The high concentrations in rice are a result of the more efficient testing process and not because the actual radioactivity concentration in rice has increased (no standard limits were exceeded in either FY2020 or FY2021).

As shown in Figure 3, there was no significant difference in internal exposure dose due to food ingestion between no regulation and under regulation in the median (the difference was at most 16.0%). In the 95th percentile, in some FYs, the effective dose under regulation was smaller than that of no regulation. In FY 2012, the difference between no regulation and under regulation was considerably large, indicating that food regulation was especially effective for those who would have received relatively high doses in the early years after the accident. In any case, the 95th percentile value is much lower than 1 mSv/y—the basis for setting the current standard limits. The ICRP states that the 95th percentile is the radiation dose for “a representative person” [35], and if the 95th percentile value is below the reference level (in this case, 1 mSv/year), the population is considered to be protected. As discussed later, in this study, dose estimation was performed by also assigning the radioactivity concentrations to the ND results; therefore, the radiation dose is probably greatly overestimated. Thus, even from conservative estimation results (i.e., overestimation), it was considered that food safety after the FDNPP accident was adequately ensured.

The values for each percentile of the internal exposure doses estimated in this study were higher than those in our previous reports [16,17]. For example, although the 95th percentile under regulation in FY 2012 was 0.0786 mSv/year in a previous study [17], it was 0.143 mSv/year in this study. In this study, we assumed a distribution for food intake (the mean was used as a fixed value in previous studies); therefore, high-intake doses could be evaluated more accurately. However, because food intake does not always correspond to a normal distribution, the food intake distribution used in this study might differ from the actual distribution, which is a limitation of this study. Furthermore, because the food intake indicated in the NHNS, Japan, is based on a one-day survey, it does not reflect habitual food intake [40]. Nevertheless, the new method using food intake distribution in this study is meaningful enough because a general dose estimation using only mean values does not reflect the variation in food intake.

Next, the relationship among the radioactivity concentrations of the monitoring test results, the cesium ingestion volume, and the food intake by food classification are discussed. Food items with moderately high radioactivity concentrations were primarily other animal meats, others (in poultries of meats), wild vegetables, and mushrooms. The food items primarily contributing to cesium ingestion were drinking water, rice, teas, and beer. Thus, there was no apparent correlation between the radioactivity concentrations in food and cesium ingestion. Specifically, other animal meats with noticeably high radioactivity concentrations were not related to cesium ingestion. In terms of food intake, drinking water, teas, rice, and various beverages were large components. Therefore, not only the radioactivity concentration in foodstuffs but also the amount of consumption of that food are major factors influencing cesium ingestion in the regulated condition.

Even if the test result was ND, the radioactivity concentration was assigned according to the LOD value and its fraction in each food classification to calculate the radiation exposure dose in this study. A moderately-high LOD was used in the monitoring tests for efficiency. The testing method requires that the LOD value is less than 25 Bq/kg or one-fifth of the standard limits, depending on the purpose [41,42]. However, a much lower LOD (i.e., approximately 0.1 Bq/kg) was adopted in Japan’s market basket study conducted for radionuclides [38]. Therefore, the radiation exposure doses estimated in this study appear to be overestimated. When comparing the radiation doses of no regulation and under regulation or identifying the differences in contribution to the exposure doses by food, as in this study, using the test results obtained from a large LOD would not pose a problem because each radiation dose was estimated under the same conditions. However, the radiation exposure dose estimated by this method might be overestimated.

This study has other limitations. There was no consideration of changes in the content of radioactive materials due to foodstuff cooking and processing (e.g., boiling, baking, and pickling). Although the effect varies depending on the cooking method [43], the cesium content of wild vegetables can be considerably reduced by removing the astringent taste (i.e., the lye) [26]. The actual radiation exposure dose would be much smaller in such a case. In addition, because random sampling of food intake was conducted independently for each food classification, this study could not reflect correlations in intake among food classifications (e.g., when a larger amount of a certain food is consumed, the intake of foods consumed in combination is also greater). Furthermore, although the current criteria for radioactive materials in foods were set by considering the radiation doses from radionuclides other than radioactive cesium, such as strontium-90, the internal exposure doses from nuclides other than radioactive cesium could not be considered. However, because radioactive cesium is the dominant radionuclide in the long term [44], its effect on the estimated radiation doses was not considerable.

## 5. Conclusions

This study verified the long-term internal radiation dose-reduction effects due to food regulations after the FDNPP accident and analyzed the contribution of each food item. In the estimation of radiation doses, a distribution was assumed for food intake as a new trial. Even in the estimation reflecting radiation doses of high intake assuming food intake distribution, the population doses (i.e., 95th percentile) were below 1 mSv/year (the basis of the current criteria) in all years with and without regulation. It was also shown that food regulation was especially effective for those who would have received moderately-high doses in the early years after the FDNPP accident. The activity concentration in a food group and the dietary intake of that food group are crucial factors in dose estimation. Food safety after the FDNPP accident has been ensured with even more careful verification. It is suggested that the data shown in this study could have provided useful support for addressing the food safety concerns of residents. As restoration efforts are underway in the affected Fukushima Prefecture, food-related industries have also resumed production, and we believe that it is important to continue to verify food safety with respect to radioactive materials.

## Figures and Tables

**Table 1:** Current standard limits for radioactive cesium (sum of ^134^Cs and ^137^Cs) for foodstuffs in Japan. The standard limits were established on 1 April 2012.

Food Category	Standard Limit as Radioactivity Concentration (Bq/kg)
Drinking water	10
Milk	50
Infant food	50
General food	100

**Table 3:** Dose coefficient (*DC*s) for total activity concentration of ^134^Cs and ^137^Cs for FY2012–FY2021 (Sv/Bq).

FY2012	FY2013	FY2014	FY2015	FY2016	FY2017	FY2018	FY2019	FY2020	FY2021
1.55 × 10^−8^	1.51 × 10^−8^	1.47 × 10^−8^	1.43 × 10^−8^	1.40 × 10^−8^	1.38 × 10^−8^	1.36 × 10^−8^	1.35 × 10^−8^	1.33 × 10^−8^	1.33 × 10^−8^

## Data Availability

Monitoring test results used in this study are available on the MHLW website.
